# Fat Stranding Associated With High-Grade Colon Adenocarcinoma: A Case Report

**DOI:** 10.7759/cureus.58815

**Published:** 2024-04-23

**Authors:** Hemangi Patel, Mahi Basra, Taraneh Honarparvar, Christopher Delange, Alejandro Biglione

**Affiliations:** 1 Sports Medicine, Nova Southeastern University Dr. Kiran C. Patel College of Osteopathic Medicine, Fort Lauderdale, USA; 2 Osteopathic Medicine, Nova Southeastern University, Clearwater, USA; 3 Internal Medicine, Wellington Regional Medical Center, Wellington, USA

**Keywords:** colon cancer, nonspecific abdominal pain, mesenteric panniculitis, high grade, pericolonic fat stranding

## Abstract

Abdominal pain is a common complaint among patients who present to the emergency department. In this setting, a CT scan of the abdomen is frequently used for diagnostic purposes. Fat stranding is an important and relevant CT finding. It is non-specific and can be associated with multiple conditions that range from benign to life-threatening. Although it may not provide the final diagnosis, it can direct the evaluating physician toward an area of concern. This case report describes an 81-year-old female presenting to the emergency department with diffuse abdominal pain. CT of the abdomen/pelvis showed mesenteric fat stranding. She was eventually diagnosed with high-grade adenocarcinoma of the colon. The radiological appearance, pathophysiology, possible etiologies, and clinical significance of fat stranding are discussed.

## Introduction

Abdominal pain is a common complaint for patients presenting in the emergency department (ED) [1]. The workup of abdominal pain frequently includes a CT of the abdomen [1]. It should ideally be performed with oral contrast. In a busy ED, waiting to obtain a CT with oral contrast may not be ideal due to time constraints. In most instances, the study is performed with intravenous (IV) contrast; however, administering IV contrast can be contraindicated in cases of renal disease or allergies to IV contrast.

Often, the only finding on the CT scan, particularly without contrast, can be fat stranding. Normal fat on CT appears as thin connective tissue that is barely visible [1,2]. Fat stranding on a CT appears as increased attenuation which can be ill-defined, reticular, linear, or reticulonodular [1,2]. It also appears with slightly increased density in the intra-abdominal fat region [1]. Typically this increased attenuation occurs around an inflamed structure. Fat generally appears dark gray on CT scans. However, when edema is present in the fat, density increases and it becomes progressively lighter and may appear with “wavy lines” [2]. Important factors that radiologists must take into consideration are the location of fat stranding, other structures involved, and any other characteristic findings on CT [3]. Fat stranding may suggest that the adjacent structure is inflamed causing edematous changes and engorgement of lymphatics [4].

The presence of fat stranding in the right upper quadrant may suggest acute cholecystitis [3,5]. In the left upper quadrant, it can indicate acute pancreatitis or pyelonephritis [3,5]. When fat stranding is present in the right lower quadrant, it can indicate acute appendicitis, omental infarct, or Crohn’s disease [3,5]. When observed in the left lower quadrant, it can indicate acute diverticulitis or bowel ischemia [3,5]. If observed in the middle of the abdomen, differential diagnoses include infectious enteritis and colitis, inflammatory bowel disease, mesenteric panniculitis, or postoperative changes [3,5]. A rare differential also includes colon cancer, as presented in the patient presented. Other differentials when present in the middle of the abdomen include infectious enteritis and colitis, inflammatory bowel disease, or postoperative changes [3,5]. This case report discusses an 81-year-old female who came to the ED with complaints of abdominal pain, was found to have fat stranding on CT, and was diagnosed with high-grade adenocarcinoma of the colon.

## Case presentation

An 81-year-old female presented to the ED with a chief complaint of abdominal pain. The abdominal pain began the day before arrival. The pain was described as diffuse, sharp, and worsening since onset with an intensity of 8/10. She denied any associated nausea, vomiting, constipation, diarrhea, or blood in the stool. She denied previous surgical procedures. She admitted she had never undergone a colonoscopy before. She had a medical history of atrial fibrillation, rheumatoid arthritis, and osteoporosis. She had a surgical history of cholecystectomy. Her home medications consisted of iron supplements, methotrexate 2.5 mg, metoprolol 25 mg, and rivaroxaban 20 mg. She denied tobacco use, alcohol consumption, or recreational drug use.

The initial vital signs included a heart rate of 58 beats per minute, blood pressure of 127/59 mmHg, respiratory rate of 17 respirations per minute, temperature of 98.2°F (36.8°C), and oxygen saturation of 98%. On physical examination, her chest was clear to auscultation. On inspection, her abdomen showed no abnormalities. On palpation, the abdomen was soft without any tenderness, there were no palpable masses, and bowel sounds were normal upon auscultation. Her admission labs are shown in Table 1.

**Table 1:** Patient’s admission labs compared to normal range.

	Patient value	Normal range
Sodium	138 mmol/L	135–145 mmol/L
Potassium	3.9 mmol/L	3.4–4.5 mmol/L
Chloride	106 mmol/L	95–108 mmol/L
Carbon dioxide	28 mEq/L	23–28 mEq/L
Blood urea nitrogen	19 mg/dL	8–21 mg/dL
Creatinine	0.95 mg/dL	0.8–1.3 mg/dL
Aspartate transaminase	12 U/L	5–30 U/L
Alanine transaminase	12 U/L	5–30 U/L
Alkaline phosphatase	67 U/L	50–100 U/L
Bilirubin	0.4 mg/dL	0.3–1.2 mg/dL
White blood cells	3.96 × 10^3^/µL	4.5–11 × 10^3^ cells/µL
Red blood cells	2.74 × 10^6^/µL	4.2–5.9 × 10^6^ cells/µL
Hemoglobin	8.3 g/dL	12–16 g/dL (females)
Hematocrit	26.7%	36–46% (females)
Platelets	128 × 10^3^ cells/µL	150–450 × 10^3^ cells/µL
Cholesterol	204 mg/dL	Less than 200 mg/dL
Triglycerides	79 mg/dL	Less than 150 mg/dL
High-density cholesterol	204 mg/dL	Above 40 mg/dL
Calcium	9.6 mg/dL	8.6–10.3 mg/dL

**Figure 1:**
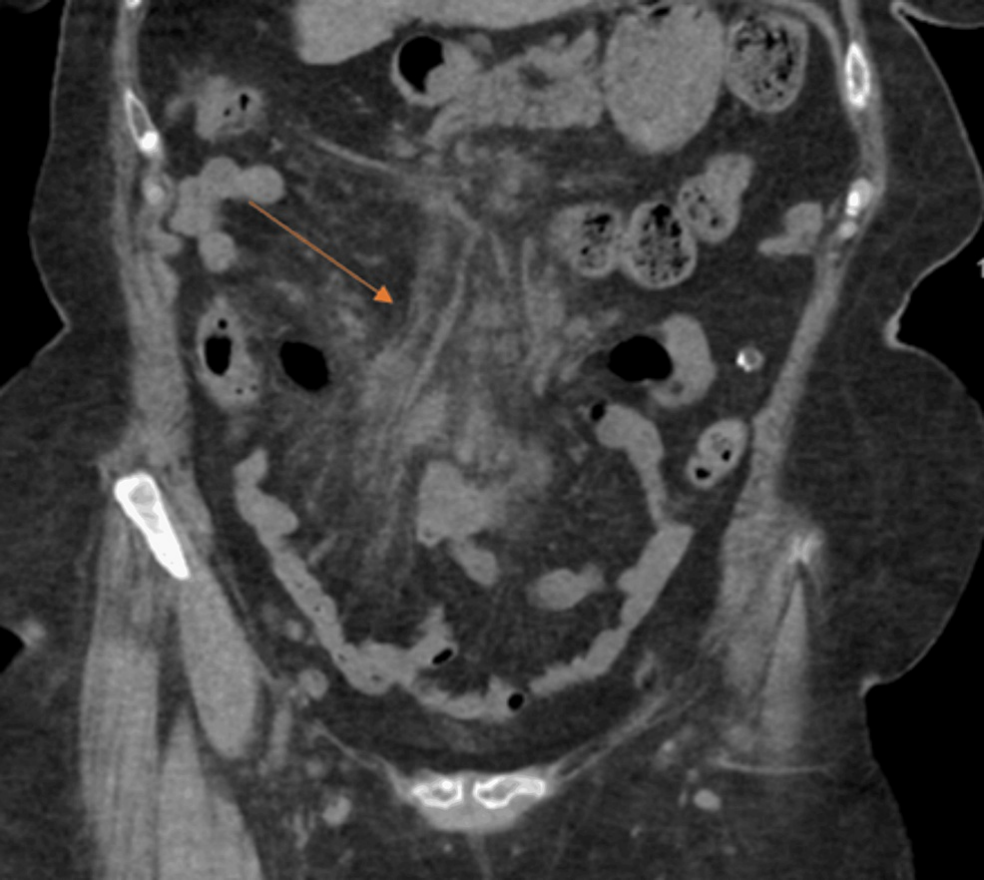
CT of the abdomen/pelvis with contrast. Fat stranding visualized (orange arrow).

The initial CT of the abdomen/pelvis without contrast showed a non-enhancing liver with no intrahepatic biliary ductal dilation. The pancreas and adrenals demonstrated normal anatomical morphology and the kidneys were within normal limits. The spleen was within normal size limits. There was no abnormal dilation of small and large bowel loops. Due to the lack of IV contrast, the evaluation of solid organs was compromised. Evidence of mesenteric fat stranding was present (Figure 1). The patient was then admitted to the hospital for further workup and management.

**Figure 2:**
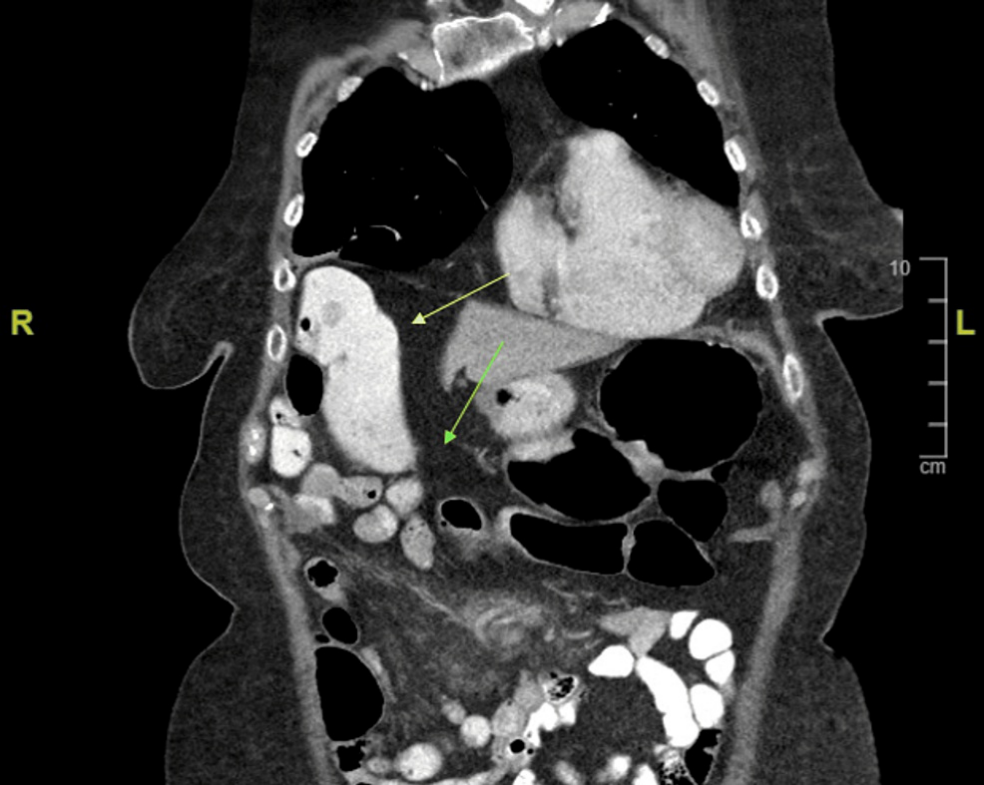
CT of the chest/abdomen/pelvis with contrast. The yellow arrow shows the initial portion of the transverse colon with contrast (opacified). The green arrow marks the mid portion of the transverse colon without contrast (not opacified) due to suspected obstruction.

Subsequently, a CT of the abdomen/pelvis with oral and IV contrast was ordered and revealed the liver was within normal limits, the gallbladder was not visualized (postoperative status), the common bile duct was not dilated, the pancreas was within normal limits, the spleen was enlarged at 15 cm with no abnormalities, and scattered colonic diverticulosis was present. There was thickening of the wall of the proximal transverse colon extending 3 cm in length with a thickness of 1.3 cm causing luminal narrowing with no evidence of bowel obstruction (Figure 2).

**Figure 3:**
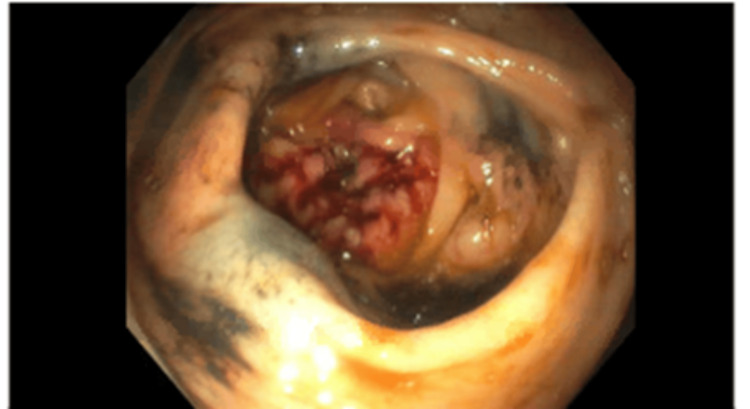
Colonoscopy findings. A fungating, infiltrative, and ulcerated completely obstructing large mass in the mid-transverse colon measured at 4 cm in length.

The patient was scheduled for a colonoscopy to clarify the CT findings of the thickened colon walls to rule out malignancy. The colonoscopy revealed a fungating, infiltrative, and ulcerated completely obstructing large mass in the mid-transverse colon (Figure 3). The mass was circumferential and measured 4 cm in length with no associated bleeding. Biopsies were taken with cold forceps for histology. The biopsy revealed high-grade invasive colon adenocarcinoma. The patient was evaluated by oncology who recommended the completion of a pretreatment workup as an outpatient including a positron emission tomography scan. She was discharged with outpatient follow-up with her primary care physician and oncology.

## Discussion

When a patient presents to the ED complaining of abdominal pain, ideally, a CT scan with IV contrast should be performed. The utilization of IV and oral contrast enhances the sensitivity and aids in distinguishing the bowel from other structures, inflammation, and detecting lesions specifically in the bowel [1]. Administration of oral contrast is not always feasible in the ED due to time constraints. The administration of IV contrast may be contraindicated in cases such as renal insufficiency or allergies [1].

**Figure 4:**
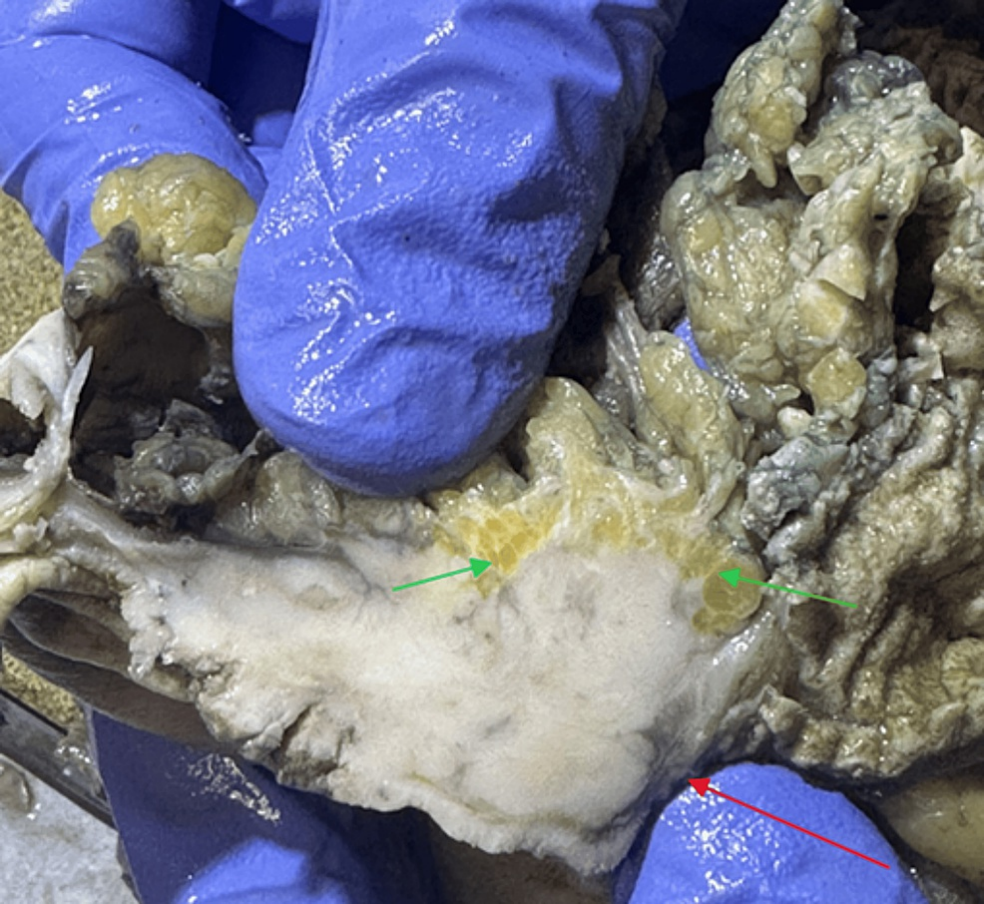
The green arrows point to the presence of fat stranding visualized as the thin white streaks adjacent to the tumor (red arrow).

**Figure 5:**
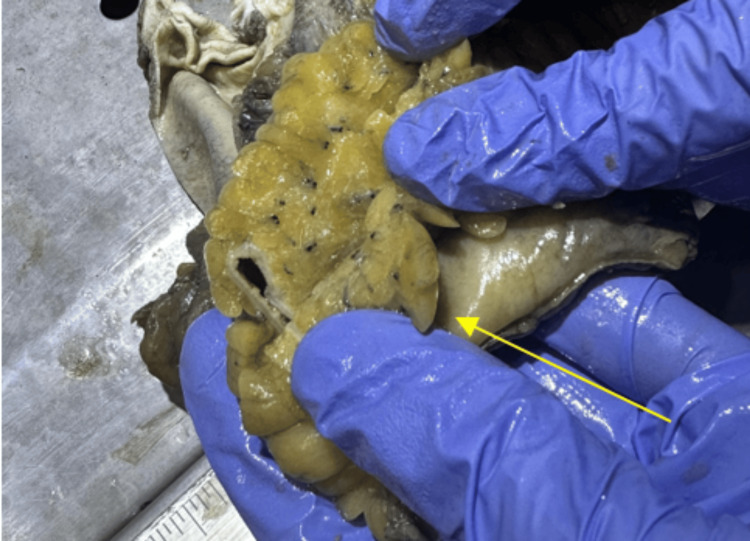
The yellow arrowing points to the appearance of normal fat in the specimen sample.

Frequently, fat stranding is the only abnormality seen on CT. It does not provide a specific diagnosis, but it can help localize where worrisome pathology may be present [6]. It can direct the eye of the examining physician toward an area of pathological concern. It is a non-specific radiologic sign in itself. As it can be present in inflammatory, infectious, and, rarely, malignant conditions, its presence should not be ignored [6,7]. Its location gives clues regarding its possible cause. When fat stranding is visualized adjacent to thickened loops of the bowel, it can be caused by acute diverticulitis, epiploic appendagitis, omental infarction, bowel ischemia, infectious enteritis or colitis, inflammatory bowel disease, acute appendicitis, or, rarely, bowel cancer [6,8]. In this case, fat stranding was present adjacent to the tumor (Figure 4). Fat stranding is seen as thin, white streaks, also described as “wavy lines” [2] (Figure 4). Normal fat appears as a dense yellow, smooth, and elastic substance [2] (Figure 5).

**Figure 6:**
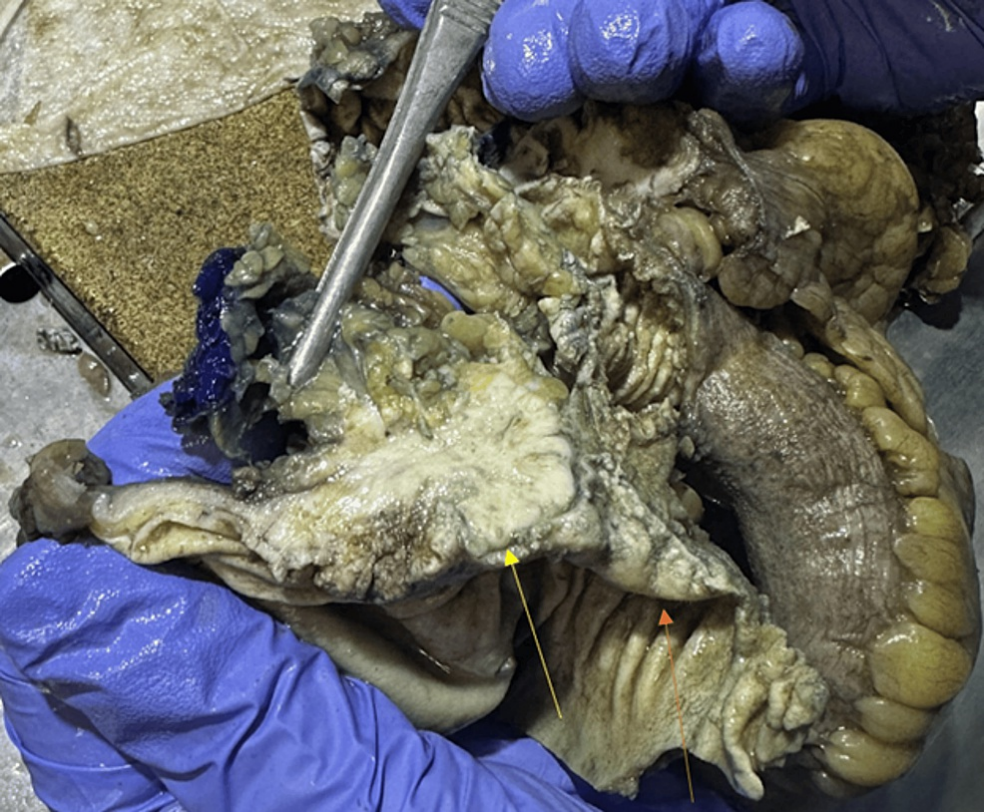
Dilated loops of the bowel (orange arrow) adjacent to the obstructing tumor (yellow arrow).

In this case, fat stranding was visualized adjacent to the colon (Figure 4) indicating a colonic pathologic process. As mentioned, fat stranding adjacent to dilated and thickened bowel loops can unusually be an indication of colon cancer [5]. In this patient, there were dilated loops of the bowel adjacent to the obstructing tumor (Figure 6). The radiological finding of fat stranding in the case eventually led to the ordering of a colonoscopy. The colonoscopy revealed a fungating, infiltrative, completely obstructing large mass in the mid-transverse colon (Figure 3). The diagnosis was then confirmed to be high-grade colon adenocarcinoma after a biopsy. The initial presence of fat stranding leading to this diagnosis emphasizes the importance of this non-specific radiological finding.

## Conclusions

Abdominal pain is a common complaint in the ED and requires a CT of the abdomen for diagnostic purposes to visualize structures. Fat stranding is a non-specific finding on CT imaging that can be associated with many benign and malignant conditions and can help focus the attention of the physician on potentially problematic regions. In this case, fat stranding was associated with colonic malignancy. The purpose of this case report is to emphasize the importance of fat stranding as a possible indirect sign of colonic malignancy in radiological studies. By doing so, it can promote proactive management of fat stranding and early diagnosis of malignancies.

## References

[1] (2024). Imaging evaluation of acute abdominal pain. https://radiologykey.com/imaging-evaluation-of-acute-abdominal-pain.

[2] (2024). Fat stranding (summary). https://radiopaedia.org/articles/fat-stranding-summary-3?lang=us#:~:text=Fat%20stranding%20is%20a%20sign,signpost%20for%20intra%2Dabdominal%20pathology.

[3] Thornton E, Mendiratta-Lala M, Siewert B, Eisenberg RL (2011). Patterns of fat stranding. AJR Am J Roentgenol.

[4] (2024). What is fat stranding?. https://medicinespecifics.com/what-is-fat-stranding/.

[5] Sharma M, Agrawal A (2008). Pictorial essay: CT scan of appendicitis and its mimics causing right lower quadrant pain. Indian J Radiol Imaging.

[6] (2024). Fat stranding (CT). https://radiopaedia.org/articles/fat-stranding-ct-1.

[7] Mehta P, Reddivari AK, Ahmad M (2020). A case report of mesenteric panniculitis. Cureus.

[8] Morimoto T, Yamada T, Miyakawa K, Nakajima Y (2018). Factors associated with pericolic fat stranding of colon cancer on computed tomography colonography. Acta Radiol Open.

